# Discovery of Novel Bioactive Tanshinones and Carnosol Analogues against Breast Cancer

**DOI:** 10.3390/cancers15041318

**Published:** 2023-02-19

**Authors:** Miguel A. González-Cardenete, Natalia González-Zapata, Lucinda Boyd, Fatima Rivas

**Affiliations:** 1 Instituto de Tecnología Química, Universitat Politècnica de València-Consejo Superior de Investigaciones Científicas, Avda. de los Naranjos s/n, 46022 Valencia, Spain; 2 Department of Chemistry, Louisiana State University, 133 Chopping Hall, Baton Rouge, LA 70803, USA

**Keywords:** breast cancer, abietane, dehydroabietylamine, catechol, ortho-quinone, phthalimide

## Abstract

**Simple Summary:**

Abietane diterpenoids are naturally occurring metabolites isolated from a large variety of plants, and many display antitumor properties. Tanshinones are abietane diterpenoids and were first isolated from *Salvia miltiorrhiza* “tanshen”, a well-known traditional Chinese medicine, which has been used extensively for the treatment of coronary heart diseases as well as inflammatory diseases. Tanshinone IIA has shown the inhibition of tumor invasion and metastasis in vitro and in vivo, while abietane carnosol found in rosemary (*Rosmarinus officinalis*) has also exhibited in vitro and in vivo anticancer activities. However, there are few reports on the pharmacological properties of the structural analogues of both molecules. The aim of this study is to synthesize and to evaluate the antitumor activities of analogues of tanshinone and carnosol. In this study, we found that the analogues were able to inhibit the proliferation of four breast cancer cell lines. Our findings show that these readily available analogues can potentially serve as the foundation of an anti-breast cancer therapeutic agent development platform.

**Abstract:**

The abietane diterpenoids ferruginol (**1**), tanshinone IIA (**3**), and carnosol (**4**) are well-known for their interesting pharmacological properties, including antitumor, similar to other natural and semisynthetic abietanes. In this study, a pair of semisynthetic C18-functionalized analogues of **3** and **4** were prepared from the commercially available (+)-dehydroabietylamine or readily obtained methyl dehydroabietate. Semisynthetic ferruginol (**1**) and some selected analogues, together with the synthesized analogues, were tested in vitro for the inhibition of proliferation in four breast cancer cell lines, SUM149, MDA-MB231, T47D, and MCF07. As a result, several tested abietane analogues decreased cell proliferation and enhanced cell death, with IC_50_ in the range 1.3–18.7 μM. This work demonstrates the antitumor activities of two tested compounds, making these molecules interesting for the development of new anticancer agents.

## 1. Introduction

Cancer is a leading cause of death worldwide. Cancer is not just a single disease, but a group of multiple diseases characterized by inappropriately controlled cell proliferation and replication, eventually resulting in the disruption of normal physiology, metabolism, or structure. Breast cancer is the most commonly diagnosed cancer in women, accounting in 2020 for 11.7% (more than 2 million) of all newly diagnosed cancer cases, and 6.9% (nearly seven hundred thousand) of all new cancer deaths worldwide [1]. Despite the tremendous progress in the past few decades, anti-cancer drug development has been considerably hampered by the limited sources of chemical scaffolds. Chemical diversity in natural products extensively attracts scientific attention to discover potential therapeutic agents such as anticancer drugs from natural sources. Nowadays, about 50 percent of commercial anticancer drugs are either natural products (NPs) or are directly derived thereof, such as vincristine, etoposide, and paclitaxel [2].

**Figure 1:**
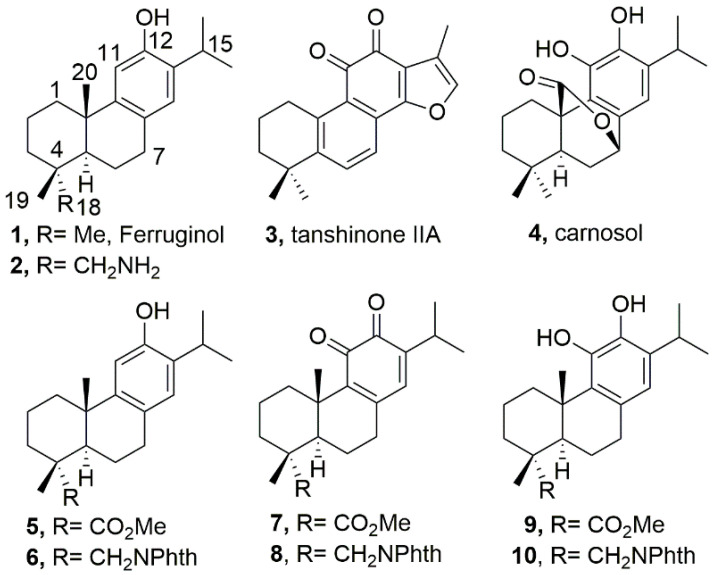
Bioactive abietanes, and tested molecules **1**–**3** and **6**–**10**.

Abietane-type diterpenoids are a family of naturally occurring metabolites isolated from a large variety of terrestrial plants that show a wide range of biological activities, including antioxidant, antimicrobial, anti-inflammatory, anti-angiogenic, anti-proliferative, and cytotoxic [3,4,5]. Many of its members have shown potent tumor growth inhibitory effects and broad anticancer activities in several human cancer cell lines, such as the aromatic abietanes dehydroabietic acid and dehydroabietylamine, also called leelamine [5,6]. The simplest phenol abietane-type diterpenoid, ferruginol (**1**) (Figure 1), has demonstrated important antitumor activities inhibiting non-small cell lung cancer growth by inducing caspase-associated apoptosis. The intraperitoneal administration of ferruginol significantly suppressed the growth of subcutaneous xenografts [7].

Traditional Chinese Medicine (TCM) has evolved over thousands of years in China and in other Asian countries for the treatment and symptoms management of a wide range of medical conditions. Recently, the National Cancer Institute (USA) has shown a renewed interest and has developed a processed library from a prototype TCM library for drug discovery by researchers worldwide [8]. Among the TCM compounds, the abietane-derived tanshinone IIA (**3**, Figure 1), an ortho-quinone, is under clinical development for cancer therapy. Tanshinones were first isolated from the roots of *Salvia miltiorrhiza* “tanshen”, a well-known TCM which has been used extensively for the treatment of coronary heart diseases as well as inflammatory diseases [9]. Tanshinones and their analogs have shown antitumor activities in various cell lines and animal models, as shown in recent studies in breast cancer [10]. Tanshinone IIA (**3**) has demonstrated the inhibition of tumor invasion and metastasis in vitro and in vivo, as well as anti-angiogenic effects [10,11]. The related abietane carnosol (**4**) (Figure 1) isolated from culinary herbs including sage, oregano, and rosemary (*Rosmarinus officinalis*) and characterized by containing a catechol group, has also shown in vitro and in vivo anticancer activity, including anti-metastatic activity [12]. Indeed, several reports demonstrate the antitumor potential in the breast cancer of carnosol (**4**) inhibiting migration, metastasis, and tumor growth via the ROS-dependent proteasome degradation of STAT3; inducing ROS-mediated beclin1-independent autophagy and apoptosis in triple-negative breast cancer; selectively inhibiting p300 histone acetyltransferase; triggering a ROS-dependent endoplasmic reticulum (ER)-stress response through the activation of three ER stress sensor pathways; and more recently, that carnosol induces p38-dependent autophagy and activates the ubiquitin-proteasome pathway [13].

Methyl 12-hydroxyabieta-8,11,13-trien-18-oate (**5**) was synthesized in our laboratory from methyl dehydroabietate as an intermediate for a semisynthesis of liquiditerpenoic acid A [14]. Phenol **5** displayed a moderate degree of activity (GI_50_ 9.7–19 μM) against two breast cancer cell lines HBL-100 and T-47D when evaluated by using the established sulforhodamine B (SRB) assay [14].

We recently extended studies with the phthalimide-ferruginol analogue **6 [15]** on its synthesis, from dehydroabietylamine, and additional antiviral experiments [16]. On having available compounds **5** and **6** [18-(phthalimid-2-yl)ferruginol] in our laboratory, derivatization was envisaged towards the corresponding ortho-quinones **7** and **8**, and the catechol **9** and **10** derivatives, which are analogues of antitumor tanshinone IIA (**3**) and carnosol (**4**), respectively.

**Scheme 1:**
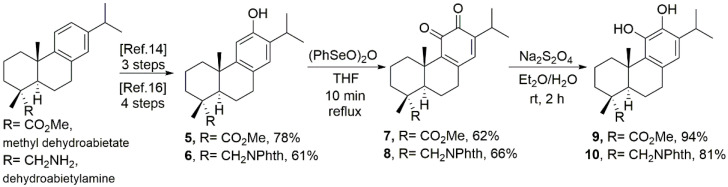
Synthetic route for tested tanshinone and carnosol analogues **7**–**10**.

Based on this background of antitumor activities of abietane-type diterpenoids, in this work, we have synthesized and evaluated the biological properties of several C18-functionalized ferruginol, tanshinone, and carnosol analogues. Thus, we report on the in vitro antitumor study of ferruginol (**1**), ferruginol analogues **2** and **6**, tanshinone analogues **7**–**8,** and carnosol analogues **9**–**10** (Figure 1 and Scheme 1) against different subtypes of human breast cancer cell lines, including the triple negative breast cancer (TNBC) models (lacking estrogen receptor, progesterone receptor, and human epidermal growth factor receptor 2 overexpression) SUM149 and MDA-MB231, and the two human hormone-dependent cell lines that represent the luminal A subtype of breast cancer, T47D and MCF07. These human breast cancer cell lines provide an excellent platform for breast cancer research in tumor progression and treatment. Based on the collected promising biological data, this family of natural products and their semisynthetic analogues are worthy of further studies due to their promising biological activities.

## 2. Materials and Methods

### 2.1. Chemistry: General Experimental Procedures

Specific rotation was measured using a 10 cm cell in a Jasco P-2000 polarimeter in DCM. NMR data were collected on a 300 MHz spectrometer. All spectra were recorded in CDCl_3_ as the solvent. Reactions were monitored via TLC using Merck silica gel 60 F254 (0.25 mm thick) plates. Compounds on TLC plates were visualized under UV light at 254 nm and via immersion in a 10% sulfuric acid solution and through heating with a heat gun. Purifications were performed via flash chromatography on Merck silica gel (230–400 mesh). Commercial reagent-grade solvents and chemicals were used as purchased. Combined organic extracts were washed with brine, dried over anhydrous MgSO4, filtered, and concentrated under reduced pressure.

The starting materials (+)-dehydroabietylamine (ca. 60%) and (−)-abietic acid >70% were purchased from Aldrich from TCI Europe, respectively. The carbon number process matching the compound to those of natural products was performed. All known compounds (**1**, **2**, **5**, and **6**) were prepared in this work following our methods, and displayed spectroscopic data, in agreement with the reported ones [14,16]. The purity of the final compound was 95% or higher. ^1^H, ^13^C, and DEPT135 spectra for compounds **7**–**10** are provided at the supplementary material (Figures S3–S14).

### 2.2. Synthesis (Scheme 1)

#### 2.2.1. Methyl 11,12-Dioxo-abieta-8,13-dien-18-oate (**7**)

Benzeneseleninic anhydride (PhSeO)_2_O (1.44 g, 4 mmol) was added to a yellow–orange solution of phenol **5** [14] (1.32 g, 4 mmol) in anhydrous THF (28 mL) and heated at reflux (bath regulated at 80 °C) for 10 min, resulting in a dark green color. The solvent was evaporated under vacuum to yield a crude product (brown), which after column chromatography on silica gel eluting with hexane/EtOAc 8:2 afforded ortho-quinone **7** (850 mg, 62%) as an amorphous brown solid: [α]^24^_D_ = −137.3 (c 1.0, CH_2_Cl_2_); ^1^H NMR (400 MHz) δ 6.38 (1H, s), 3.67 (s, 3H), 2.88 (1H, sept., *J* = 6.8), 2.78–2.75 (1H, m), 2.49–2.42 (2H, m), 2.00 (1H, dd, *J* = 12.4, 1.6), 1.72–1.60 (5H, m), 1.40–1.30 (1H, m), 1.24 (3H, s), 1.23 (3H, s), 1.08 (3H, d, *J* = 6.8), 1.07 (3H, d, *J* = 6.8); ^13^C NMR (100 MHz) δ_C_ 180.9 (s), 179.9 (s), 178.8 (s), 148.0 (s), 146.9 (s), 144.1 (s), 137.6 (d), 52.0 (q), 47.6 (s), 45.5 (d), 37.4 (s), 36.7 (t), 35.2 (t), 33.2 (t), 26.9 (d), 21.4 (q), 21.4 (q), 20.8 (t), 20.2 (q), 18.0 (t), 16.5 (q); HRMS (ESI) m/z 345.2062 [M+H]+, calcd for C_21_H_29_O_4_: 345.2066; Anal. calcd. for C_21_H_28_O_4_: C, 73.2; H, 8.2; Found: C, 73.1; H, 8.1.

#### 2.2.2. 11,12-Dioxo-N,N-(phthaloyl)dehydroabietylamine (**8**)

Benzeneseleninic anhydride (PhSeO)_2_O (295 mg, 0.82 mmol) was added to a solution of phenol **6** [16] (352 mg, 0.82 mmol) in anhydrous THF (6 mL) and heated at reflux (bath regulated at 80 °C) for 10 min under a nitrogen atmosphere. The solvent was evaporated under vacuum (bath 30 °C) to yield a crude product (brown-red semisolid), which was suspended in hexane/EtOAc 8:2 and chromatographed onto silica gel, eluting with hexane/EtOAc 8:2 to afford ortho-quinone **8** (242 mg, 66%) as an olive–green foam: [α]^24^_D_ -140.5 (c 0.7, CH_2_Cl_2_); ^1^H NMR (400 MHz) *δ* 7.85–7.83 (2H, m), 7.73–7.71 (2H, m), 6.41 (1H, s), 3.68 (1H, d, *J* = 14.0), 3.43 (1H, d, *J* = 14.0), 2.87 (1H, sept., *J* = 6.8), 2.72–2.68 (1H, m), 2.62–2.58 (1H, m), 2.55–2.45 (2H, m), 2.28–2.23 (1H, m), 1.65–1.55 (2H, m), 1.52–1.44 (2H, m), 1.27 (3H, s), 1.18 (1H, d, *J* = 10.8), 1.08 (6H, d, *J* = 6.8), 1.02 (3H, s), 0.97–0.89 (1H, m); ^13^C NMR (100 MHz) *δ*_C_ 181.2 (s), 180.2 (s), 2 × 169.4 (s), 148.4 (s), 146.7 (s), 144.5 (s), 137.8 (d), 2 × 134.0 (d), 132.0 (s), 2 × 123.3 (d), 48.7 (t), 45.8 (d), 39.3 (s), 38.1 (s), 36.3 (t), 35.3 (t), 33.6 (t), 26.8 (d), 21.4 (q), 21.3 (q), 20.9 (q), 19.4 (q), 18.5 (t), 18.0 (t); HRMS (ESI) m/z 446.2339 [M+H]+, calcd for C_28_H_32_NO_4_: 446.2331; Anal. calcd. for C_28_H_31_NO_4_: C, 75.5; H, 7.0; N, 3.1; Found: C, 75.3; H, 7.0; N, 3.4.

#### 2.2.3. Methyl 11,12-Dihydroxy-dehydroabietate (**9**)

To a solution of quinone **7** (710 mg, 2.06 mmol) in diethyl ether (75 mL) was added a solution of aqueous Na_2_S_2_O_4_ (10%, 75 mL), and the mixture was stirred vigorously (600 rpm) until the brown–greenish solution became yellowish (about 2 h). Then, the organic phase was separated, and the aqueous phase was extracted once with diethyl ether (20 mL). The combined organic extracts were washed with brine (15 mL), dried, and concentrated to yield the crude product as a light pale foam. The crude was dissolved in hexane/EtOAc 8:2 and a bit of DCM, and chromatographed on silica, eluting with hexane/EtOAc 8:2 to afford catechol **9** (670 mg, 94%) as an amorphous green solid: [α]^23^_D_ = +59.3 (c 1.2, CH_2_Cl_2_); ^1^H NMR (400 MHz) δ 6.42 (1H, s), 5.77 (1H, s), 4.70 (1H, br s), 3.67 (s, 3H), 3.16–3.12 (1H, m), 2.99 (1H, sept., *J* = 6.8), 2.86–2.74 (2H, m), 2.22 (1H, dd, *J* = 12.0, 1.2), 1.80–1.55 (5H, m), 1.45–1.37 (1H, m), 1.36 (3H, s), 1.28 (3H, s), 1.24 (3H, d, *J* = 6.8), 1.22 (3H, d, *J* = 6.8); ^13^C NMR (100 MHz) δ_C_ 179.5 (s), 143.2 (s), 137.9 (s), 132.4 (s), 131.8 (s), 129.6 (s), 117.2 (d), 51.9 (q), 48.4 (s), 47.0 (d), 38.5 (s), 36.5 (t), 35.9 (t), 31.8 (t), 27.2 (d), 22.8 (q), 22.5 (q), 22.3 (t), 20.3 (q), 18.6 (t), 16.8 (q); HRMS (ESI) m/z 369.2033 [M+Na]^+^, calcd for C_21_H_30_O_4_Na: 369.2042; Anal. calcd. for C_21_H_30_O_4_: C, 72.8; H, 8.7; Found: C, 73.1; H, 8.9.

#### 2.2.4. 11,12-Dihydroxy-N,N-(phthaloyl)dehydroabietylamine (**10**)

To a solution of quinone **8** (220 mg, 0.5 mmol) in diethyl ether (50 mL) was added a solution of aqueous Na_2_S_2_O_4_ (10%, 50 mL), and the mixture was stirred vigorously (600 rpm) until the brown–green solution became yellowish (about 2 h). Then, the organic phase was separated and the aqueous phase was extracted once with diethyl ether (10 mL). The combined organic extracts were washed with brine (10 mL), dried, and concentrated to yield the crude product as an orange semisolid, which when triturated with hexane, yielded a yellow solid. The crude (240 mg) was dissolved in hexane/EtOAc 7:3 and a bit of DCM, and chromatographed on silica, eluting with hexane/EtOAc 7:3 to afford catechol **10** (180 mg, 81%) as a greenish semisolid, which when triturated with hexane, yielded a light green–white solid: [α]^24^_D_ = +1.5 (c 0.8, CH_2_Cl_2_); ^1^H NMR (400 MHz) *δ* 7.83–7.81 (2H, m), 7.71–7.69 (2H, m), 6.46 (1H, s), 5.71 (1H, s), 4.71 (1H, br s), 3.67 (1H, d, *J* = 14.0), 3.56 (1H, d, *J* = 14.0), 3.08–3.04 (1H, m), 3.00–2.80 (3H, m), 2.12 (1H, dd, *J* = 12.4, 6.0), 1.72–1.55 (4H, m), 1.45–1.40 (3H, m), 1.38 (3H, s), 1.24 (3H, d, *J* = 6.8), 1.22 (3H, d, *J* = 6.8), 1.05 (3H, s); ^13^C NMR (100 MHz) *δ*_C_ 2 × 169.4 (s), 142.9 (s), 138.0 (s), 2 × 133.9 (d), 132.8 (s), 132.0 (s), 131.7 (s), 129.7 (s), 2 × 123.2 (d), 117.3 (d), 49.2 (t), 47.7 (d), 39.6 (s), 39.3 (s), 36.3 (t), 36.1 (t), 31.9 (t), 27.2 (d), 22.7 (q), 22.5 (q), 21.3 (q), 19.7 (t), 19.6 (q), 18.6 (t); HRMS (ESI) m/z 470.2118 [M+Na]+, calcd for C_28_H_33_NNaO_4_: 470.2307; Anal. calcd. for C_28_H_33_NO_4_: C, 75.1; H, 7.4; N, 3.1; Found: C, 74.7; H, 7.2; N, 3.3.

### 2.3. Antitumor Assay

#### 2.3.1. Cell Culture and Cell Viability Assays

Cell line models as TNBC models (MDA-MB231, SUM19), ER+ breast cancer (MCF-7, T-47D), and non-tumorigenic cell line BJ were purchased from the American Type Culture Collection (ATCC, Manassas, VA, USA), except for SUM149, which was obtained from Asterand (Detroit, MI, USA). Cells were maintained under suggested media conditions, supplemented with fetal bovine serum in a humidified atmosphere of 5% CO_2_ at 37 °C. Our cell lines are authenticated every six months using the Cell Check 9 service (Idexx BioResearch, Westbrook, ME, USA). Cells were tested for mycoplasma prior to use (MycoAlert detection Kit Lonza LT07-318) and discarded if they tested positive. A cell proliferation assay was performed using the CellTiterGlo (CTG, Promega Corp., Madison, WI, USA) luminescent cell viability assay kit. MDA-MB231 cells were seeded into 96-well plates at concentrations experimentally determined to ensure logarithmic growth during the duration of the experiment and to prevent adverse effects on cell growth by DMSO exposure. The plates were incubated 12 h before treatment. Stock solutions of test compounds (10 mM in DMSO) in nine three-fold serial dilutions were dispensed to have compounds in a concentration range from 100 μM to 0.75 μM. The final concentration of DMSO was 0.3% (*v*/*v*) in each well. The positive control used was staurosporine (1 μM) and a toxic quinolone generated inhouse, as kill-all controls, and the negative control used was cholesterol. Staurosporine is a potent alkaloid inducer of various cell death modalities, but primarily apoptosis, making it a reliable positive control for viability assays [17]. The plates were incubated for 72 h and then quenched with CTG at 50 μL per well at RT. Plates were then incubated at RT for 20 min and centrifuged at 1000 rpm for 1 min. Luminescence was read on a CLARIOstar Plus plate reader (BMG LabTech, Ortenberg, Germany). The cell viability assay was also performed in the non-neoplastic cell line BJ, which was incubated with compounds, as mentioned in cell viability assay (CTG). The mean luminescence of each experimental treatment group was normalized as a percentage of the mean intensity of untreated controls. EC_50_ values were calculated from dose-response curve-fitting via non-linear regression using GraphPad Prism 9.0 (GraphPad Software, San Diego, CA, USA) (Figure S1). A therapeutic index (TI) between normal and tumor cell lines can be determined (EC_50_ non-neoplastic cell line BJ)/(EC_50_ cancer cell line); see Figure S2 in Supplementary Information for a graphic representation.

Propidium iodide (PI) assay was performed to complement the viability assay. The assay was conducted with MDA-MB231 cancer cell models seeded in 48-well plates (Clear, Corning#3548) and cultured for 12 h, then treated for 48 h. Cells were fixed with cold methanol, and stained (0.4% PI, Sigma-Aldrich, Co., St. Louis, MO, USA). Fluorescence was measured and analyzed using Cytation 1 Image Reader (BioTek, Winooski, VT, USA). Cell viability was calculated/plotted as the percentage of surviving cells after treatment relative to the vehicle. For qualitative analysis and visualization, doubling staining was performed using Hoechst dye to stain nuclei.

All experiments were conducted with technical replicates and at least 3 biological replicas to ensure the robustness and reproducibility of the assays.

#### 2.3.2. Cell Morphology

Data were collected from nine different fields in fluorescent images acquired using 5× and 10× objective with a Leica DMi1 Microscope (Leica microsystems, Wetzlar, Germany). MDA-MB231 cells were imaged with a vehicle as the negative control, staurosporine as the positive control (2 µM), or compounds with results shown in representative images for 72 h.

#### 2.3.3. Antioxidant Assay

An antioxidant evaluation of the compounds was performed using the Ferric Reducing Antioxidant Power (FRAP) Assay Colorimetric Kit (ab234626, Abcam, Cambridge, UK) following the manufacturers’ instructions. Briefly, FRAP reagents were mixed with compounds at 100 μM in 96-well plates, and then reacted for 10 min at 37 °C in the dark. Ascorbic acid was provided as the positive control (1mM) [18]. After incubation, the absorbance was read using a microplate reader (CLARIOstar Plus, BMG LabTech, Ortenberg, Germany) at 594 nm. FRAP [19] was quantified using a reagent calibration curve (Y = 0.039X + 0.002, R^2^ = 0.9978) and calculated as nmol Ferrous equivalents.

## 3. Results

### 3.1. Chemistry

Compounds **1** (ferruginol) and **2** (12-hydroxydehydroabietylamine) (Figure 1) were synthesized following our reported procedures, starting from the commercially available (+)-dehydroabietylamine [16,20]. The analogues **7–10** were prepared from either methyl dehydroabietate through intermediate methyl 12-hydroxyabieta-8,11,13-trien-18-oate (**5**) or dehydroabietylamine through intermediate 18-(phthalimid-2-yl)ferruginol (**6**), which were readily in three and four synthetic steps with 78% and 61% overall yields, respectively (Scheme 1). Having in hand the intermediates **5** and **6**, those were subjected to oxidation in C11 and C12 with benzeneseleninic anhydride, (PhSeO)_2_O, in boiling THF for a short period of time (10 min), as reported for the oxidation of ferruginol itself [21]. Thus, the ortho-quinones **7** and **8** were obtained at 62% and 66% yields, respectively. Next, a mild reduction reaction on both quinones was performed with sodium dithionite, Na_2_S_2_O_4_, to afford the corresponding catechols **9** and **10**, at 94% and 81% yields, respectively (Scheme 1).

### 3.2. Biology

**Figure 2:**
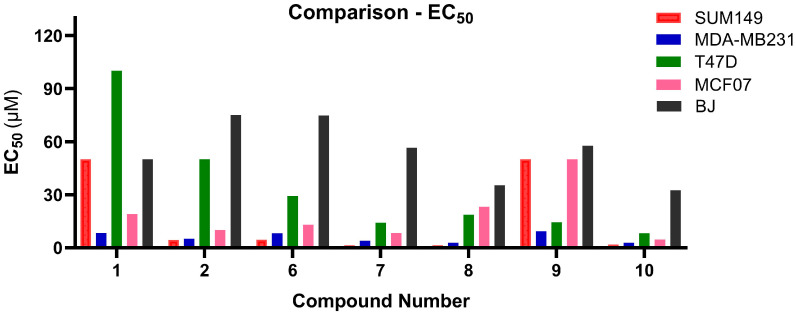
Column bar graphic to compare activities of compounds in the different cell lines.

The experimental models (cancer cell lines: SUM149, MDA-MB231, T47D, MCF07) for the breast cancer studies enabled the evaluation of compounds **1**–**2** and **6**–**10** (Table 1, Figure 2), which indicate that the compounds inhibit cell proliferation, as evaluated with the CellTiterGlo assay [22]. Interestingly, the compounds display stronger antiproliferative effects against TNBC models (MDA-MB231 and SUM19). Compound **2**, **8**, and **10** demonstrated the most promising results, as the therapeutic index was observed when compared to the non-tumorigenic cell line BJ response.

**Figure 3:**
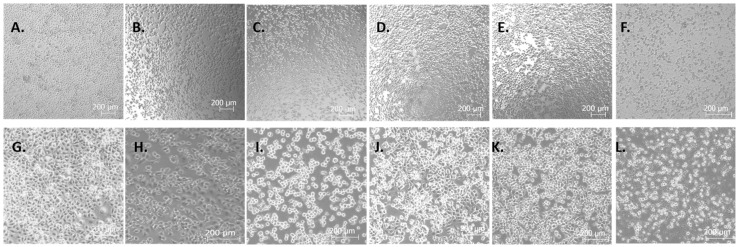
Morphological changes of MDA-MB-231 cells when treated with compounds or vehicle. At 5×: (**A**) DMSO, (**B**) **8** (5 µM), (**C**) **8** (10 µM), (**D**) **2** (5 µM), (**E**) **2** (10 µM), (**F**) STS (2 µM). At 10×: (**G**) DMSO, (**H**) **8** (5 µM), (**I**) **8** (10 µM), (**J**) **2** (5 µM), (**K**) **2** (10 µM), (**L**) STS (2 µM). STS: Staurosporine.

**Figure 4:**
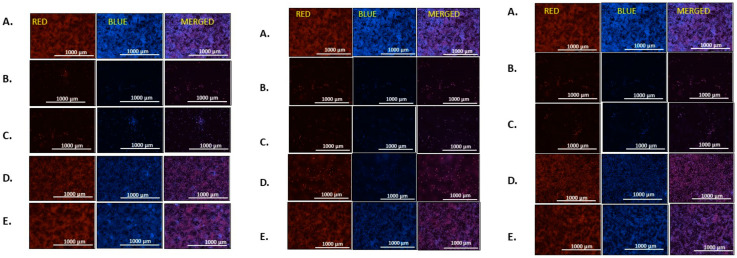
Analysis of apoptosis using propidium iodide (red) staining of MDA-MB-231 cells when treated with compound **2** (**left**), **8** (**center**), and **10** (**right**) at different doses, or vehicle for 48 h. Counterstain Hoersch (blue) was also used to image at 5×: (**A**) DMSO, (**B**) STS (2 µM), (**C**) compound at 50 µM, (**D**) compound at 25 µM, (**E**) compound at 12 µM. STS: Staurosporine.

Cell morphology representative images captured the process of apoptosis (Figure 3), which was further validated with the double staining of propidium iodide with Hoechst nuclear staining, which suggested dose-dependent cell death (Figure 4). The exact mechanism of action of these compounds is yet to be identified. Because a differential therapeutic index was observed, it was assumed that an organized inhibition of proliferation (cytotoxicity) and/or cell death was implicated here. Different cell death modalities are possible, and a stepwise process is favored. Brightfield images indicate the morphologic hallmarks of apoptosis after compound treatment, such as a loss of cell–cell contact, where the cell surface is altered, leading to the rounding of the structure and cell shrinkage (Figure 3G vs. I). Furthermore, the PI assay can provide further dose-dependency effects of compound treatment, leading to cell death (Figure 4C vs. D). Compound **8** clearly indicates this dependency at 25 μM. While extended periods are required to induce cell death, these compounds indicate a promising platform to develop new molecules, with selectivity towards unhealthy proliferative tissue.

**Figure 5:**
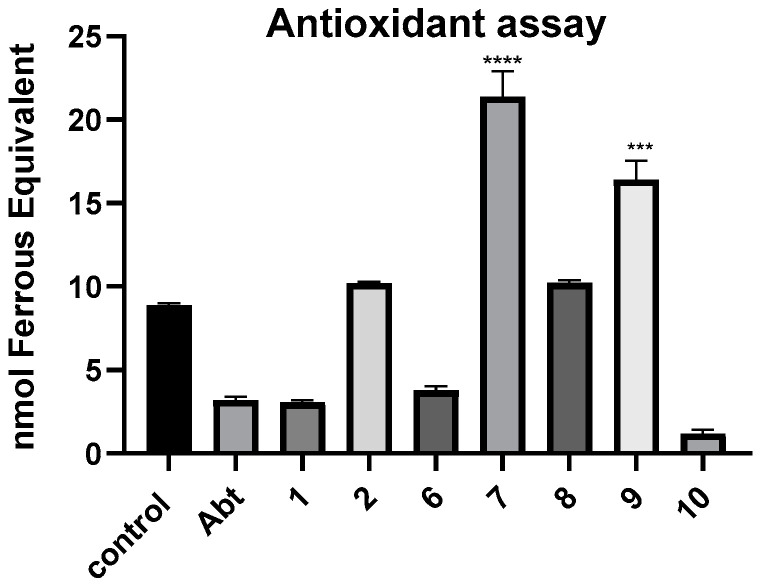
Antioxidant capacity evaluation via FRAP. Compounds were evaluated for antioxidant capacity at 100 μM (Abt = dehydroabietylamine). Values are expressed as mean ± SE (*n* = 3). Error bars indicate standard errors between control and individual compounds **7** and **9**, respectively, *** *p* < 0.001, **** *p* < 0.0001.

Previous studies have indicated a correlation between the anti-proliferative and antioxidant activities of natural products [23,24]; therefore, these compounds were tested using a ferric ion reducing antioxidant power (FRAP) assay [19,25]. Compounds **2** and **8** showed similar antioxidant potentials to the positive controls, while both compounds **7** and **9** displayed significant antioxidant potential (Figure 5).

## 4. Discussion

We reported in 2015 that several abietane-type diterpenoids exhibited antiproliferative activity in several cell models of breast cancer with EC_50_ < 50 μM, including dehydroabietylamine, ferruginol (**1**), and a catechol, carnosol (**4**) (Figure 1), and sulfonamide derivatives of dehydroabietylamine [22]. In that work, ferruginol (**1**) demonstrated cell cycle arrest at G0/G1 (50.76%; positive control 43%, negative control 48.36%) and the inhibition of cell cycle progression at the G2/M phase in 19% in the model MDA-MB231. We probed that poly-(ADP-ribose) polymerase (PARP) was involved in programmed cell death due to compound **1**. In this study, it was envisioned that related catechol-containing analogues of carnosol (**4**) and their corresponding ortho-quinone precursors, analogues of tanshinones, may also possess certain antitumor activities, with a potential relationship with the antioxidant potential of the molecules due to the ring C substitution.

The most potent compound in general, in all tested breast cancer models, was compound **10**, which was particularly potent in the TNBC models, though the most potent compound for the cell line SUM149 was compound **7** with IC_50_ of 1.3 μM (Table 1). On comparing phenols **1** and **2**, it can be deduced that the presence of the amino group increased the potency towards all models with low cytotoxicity. The same tendency happens with the ferruginol analogue **6**, containing a phthalimide group at C18. The tanshinone analogues **7** and **8** displayed important activities, with the cell line T47D being the most chemoresistant, with IC_50_ from 14.1 to 18.7 μM, respectively. The conversion of quinone **7** to its corresponding catechol **9** removed the activity towards cell lines SUM149 and MCF07. The analogous conversion of quinone **8** to catechol **10** only affected to the potency against the MCF07 cell line, which was reduced by 50% (from 2.3 to 4.6 μM), and the potency against the T47D cell line, which in this case was increased (from 18.7 to 8.2 μM) (Table 1). Compounds **5** and **9** have been studied recently by Nakagawa-Goto and co-workers with the sulforhodamine B assay in the cell lines MDA-MB231 (**5**, 18.6 μM; **9**, 2.7 μM) and MCF-7 (**5**, 5.9 μM; **9**, 2.8 μM) [26]. Our results for compound **9** are somewhat different—particularly for MCF07, if we compare them—but we can conclude similarly the importance of two oxygenated functional groups at C11 and C12.

With respect to the antioxidant activity (Figure 5), it can be deduced that generally, the greater the reducing antioxidant potency, the more antiproliferative activity is found, except for compound **10**, which does not show a direct correlation. More mechanist studies are required to understand the driving factors involved in their mode of action, and these are outside the aim of this study. The direct correlation of antioxidant activity with antiproliferative action is challenging since both these processes are multifactorial due to various pathways being involved. However, compounds with readily accessible electrophilic sites can modulate the cellular response. While inducing the antioxidant program in the cell, a disbalance of reactive oxygen species (ROS) can ultimately render the cells as unable to survive [27,28,29]. Abietic acid was able to induce the overexpression of total antioxidants in the MCF-7 cells [30].

## 5. Conclusions

In summary, we have demonstrated that readily accessible tanshinone and carnosol analogues are endowed with relevant anti-breast cancer properties, especially in the TNBC models. They are accessed in short synthetic sequences at 30–50% overall yield from either the easily available methyl dehydroabietate or the commercial (+)-dehydroabietylamine. Methyl dehydroabietate is obtained from commercial chiral (–)-abietic acid via methylation and aromatization [14]. In conclusion, while the overall activities of these compounds are moderate, further mechanistic studies are warranted.

## Figures and Tables

**Table 1:** Cytotoxicity (IC_50_ in µM) semisynthetic abietanes **1–2** and **6–10** after 72 h of incubation.

Compound	SUM149	MDA-MB231	T47D	MCF07	BJ	TI
**1**	>50	8.3 ± 1.4	>100	19.0 ± 1.5	>50	1–6
**2**	4.4 ± 0.3	5.1 ± 0.6	>50	10.0 ± 1.5	75.0 ± 6.2	1–17
**6**	4.4 ± 0.2	8.2 ± 0.5	29.2 ± 1.5	13.0 ± 0.6	74.8 ± 5.3	2–17
**7**	1.3 ± 0.6	3.9 ± 0.8	14.1 ± 0.5	8.3 ± 0.9	56.5 ± 6.1	4–44
**8**	1.5 ± 0.4	2.8 ± 0.4	18.7 ± 0.3	2.3 ± 1.1	35.4 ± 5.0	1–24
**9**	>50	9.4 ± 0.4	14.4 ± 0.6	>50	57.6 ± 3.0	1–4
**10**	1.8 ± 0.2	2.8 ± 0.2	8.2 ± 0.4	4.6 ± 0.6	32.5 ± 5.0	4–18

Cells were treated with compounds for 72 h. Cytotoxicity was determined as the IC_50_ values calculated from the percentage of viable cells remaining at 72 h, measured with the CellTiter-Glo reagent. The IC_50_ values are indicated as the mean ± SD of at least three independent experiments. TI: Therapeutic index = (EC50 non-neoplastic cell line BJ)/(EC50 cancer cell line).

## Data Availability

The data are available in the manuscript and in the supporting information of this article.

## Supplementary Materials

The following supporting information can be downloaded at: https://www.mdpi.com/article/10.3390/cancers15041318/s1, Figure S1: Diagrams of viability versus concentration; Figure S2: Normalized therapeutic index and statistical comparison; Figure S3: ^1^H NMR spectrum of **7**; Figure S4. ^13^C NMR spectrum of **7**; Figure S5. DEPT135 spectrum of **7**; Figure S6. ^1^H NMR spectrum of **8**; Figure S7. ^13^C NMR spectrum of **8**; Figure S8. DEPT135 spectrum of **8**; Figure S9. ^1^H NMR spectrum of **9**; Figure S10. ^13^C NMR spectrum of **9**; Figure S11. DEPT135 spectrum of **9**; Figure S12. ^1^H NMR spectrum of **10**; Figure S13. ^13^C NMR spectrum of **10**; Figure S14. DEPT135 spectrum of **10**.
