# Supplementary material for: Discovery of Novel Bioactive Tanshinones and Carnosol Analogues against Breast Cancer
Source: Cancers (Basel). 2023 Feb 19;15(4):1318. doi: 10.3390/cancers15041318 (PMC9953967; doi:10.3390/cancers15041318)
Supplement: Supplementary file 1 [file cancers-15-01318-s001.zip › cancers-2219077-supplementary.pdf]

Supplementary Information

Discovery of novel bioactive tanshinones and carnosol analogues against breast cancer

Miguel A. González-Cardenete [1,*], Natalia González-Zapata [1], Lucinda Boyd [2] and Fatima Rivas [2]

[1] Instituto de Tecnología Química, Universitat Politècnica de València-Consejo Superior de Investigacio-nes Científicas, Avda. de los Naranjos s/n, 46022 Valencia, Spain
[2] Department of Chemistry, Lousiana State University, 133 Chopping Hall, Baton Rouge, LA 70803, USA

Email: migoncar@itq.upv.es

Contents

Copies of ^{1}H NMR , and ^{13}C NMR spectra for compounds **7-10**:

-Diagrams of cell viability versus concentration……………………………...Figure S1

-Normalized therapeutic index and statistical comparison……………………Figure S2

-^{1}H NMR spectrum of **7**…………...……...………………………………Figure S3

-^{13}C NMR spectrum of **7**………..…..………………………………………Figure S4

-DEPT135 spectrum of **7**…………………………………………………….Figure S5

-^{1}H NMR spectrum of **8**…………...……...………………………………Figure S6

-^{13}C NMR spectrum of **8**………..…..………………………………………Figure S7

-DEPT135 spectrum of **8**…………………………………………………….Figure S8

-^{1}H NMR spectrum of **9**…………...……...………………………………Figure S9

-^{13}C NMR spectrum of **9**………..…..………………………………………Figure S10

-DEPT135 spectrum of **9**…………………………………………………….Figure S11

-^{1}H NMR spectrum of **10**……..……...……...……………………………Figure S12

-^{13}C NMR spectrum of **10**………..…..…………………………………...…Figure S13

-DEPT135 spectrum of **10**……………………………………………………..…Figure S14

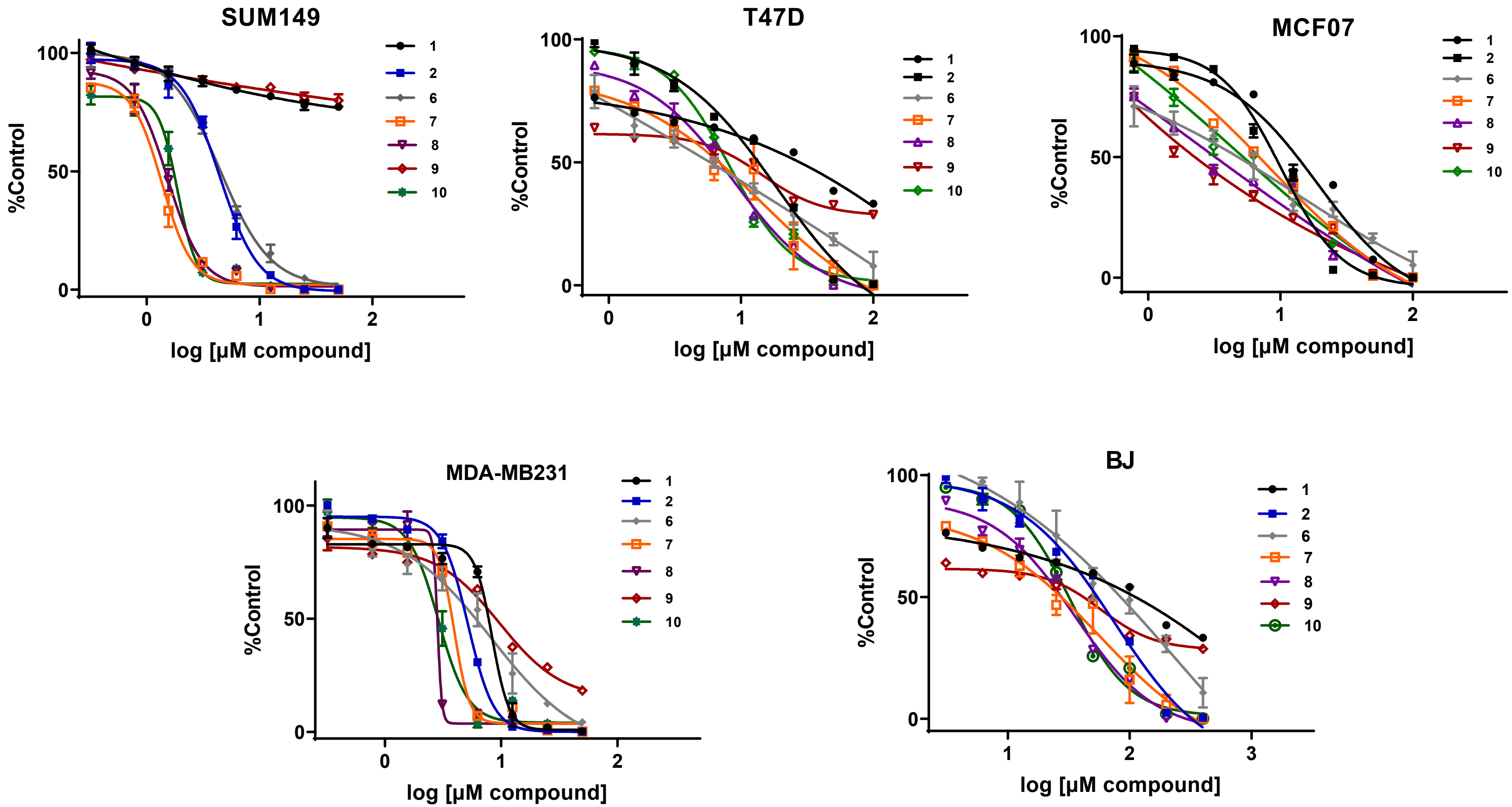

Figure S1. Diagrams of cell viability versus concentration.

SUM149	MDA-MB231	T47D	MCF07	BJ	#
>50	8.3± 1.4	>100	19.02± 1.5	>50	**1**
4.4± 0.27	5.1± 0.62	>50	10± 1.50	75± 6.2	**2**
4.45± 0.2	8.22± 0.47	29.21±1.5	13.04± 0.61	74.77± 5.3	**6**
1.3± 0.64	3.95± 0.8	14.1±0.51	8.29± 0.93	56.48± 6.1	**7**
1.5± 0.38	2.8± 0.43	18.69±0.3	2.3± 1.1	35.42± 5.0	**8**
>50	9.4± 0.45	14.4±0.62	>50	57.64± 3.0	**9**
1.8± 0.2	2.8± 0.16	8.17± 0.44	4.6± 0.64	32.49± 5.0	**10**

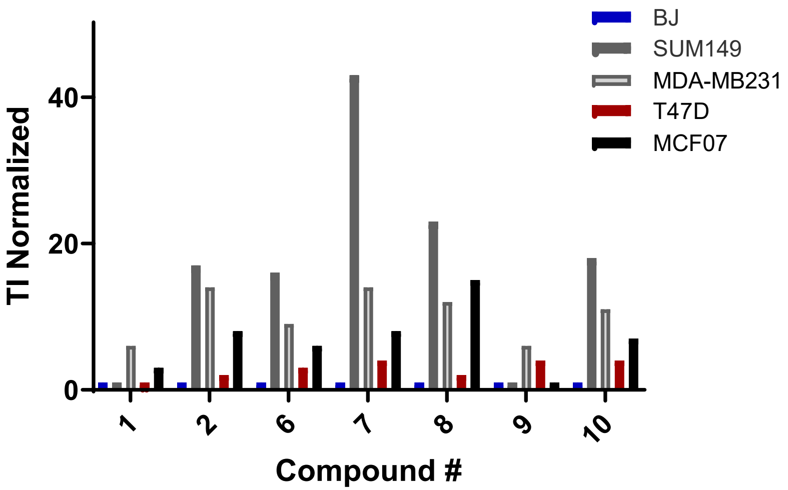

SUM149	MDA-MB231	T47D	MCF07	#
1	6	0.5	2.6	**1**
17	14	1.5	7.5	**2**
16	9	2.5	5.7	**6**
43	14	4	6.7	**7**
23	12	1.9	15	**8**
1.1	6	3.9	1.1	**9**
18	11	3.9	7	**10**

Normalized Therapeutic index = (EC50 non-neoplastic cell line BJ)/(EC50 cancer cell line).

Column B SUM149
vs. vs.
Column A BJ
Paired t test
P value 0.0252
P value summary *
Significantly different (P < 0.05)? Yes
One- or two-tailed P value? Two-tailed
t, df t=2.961, df=6
Number of pairs 7

Column C MDA-MB231
vs. vs.
Column A BJ
Paired t test
P value 0.0004
P value summary ***
Significantly different (P < 0.05)? Yes
One- or two-tailed P value? Two-tailed
t, df t=7.222, df=6
Number of pairs 7

Column D T47D
vs. vs.
Column A BJ
Paired t test
P value 0.0068
P value summary **
Significantly different (P < 0.05)? Yes
One- or two-tailed P value? Two-tailed
t, df t=4.044, df=6
Number of pairs 7

Column E MCF07
vs. vs.
Column A BJ
Paired t test
P value 0.0131
P value summary *
Significantly different (P < 0.05)? Yes
One- or two-tailed P value? Two-tailed
t, df t=3.482, df=6
Number of pairs 7

Figure S2. Normalized Therapeutic index and graphic corresponding to the statistical significance.

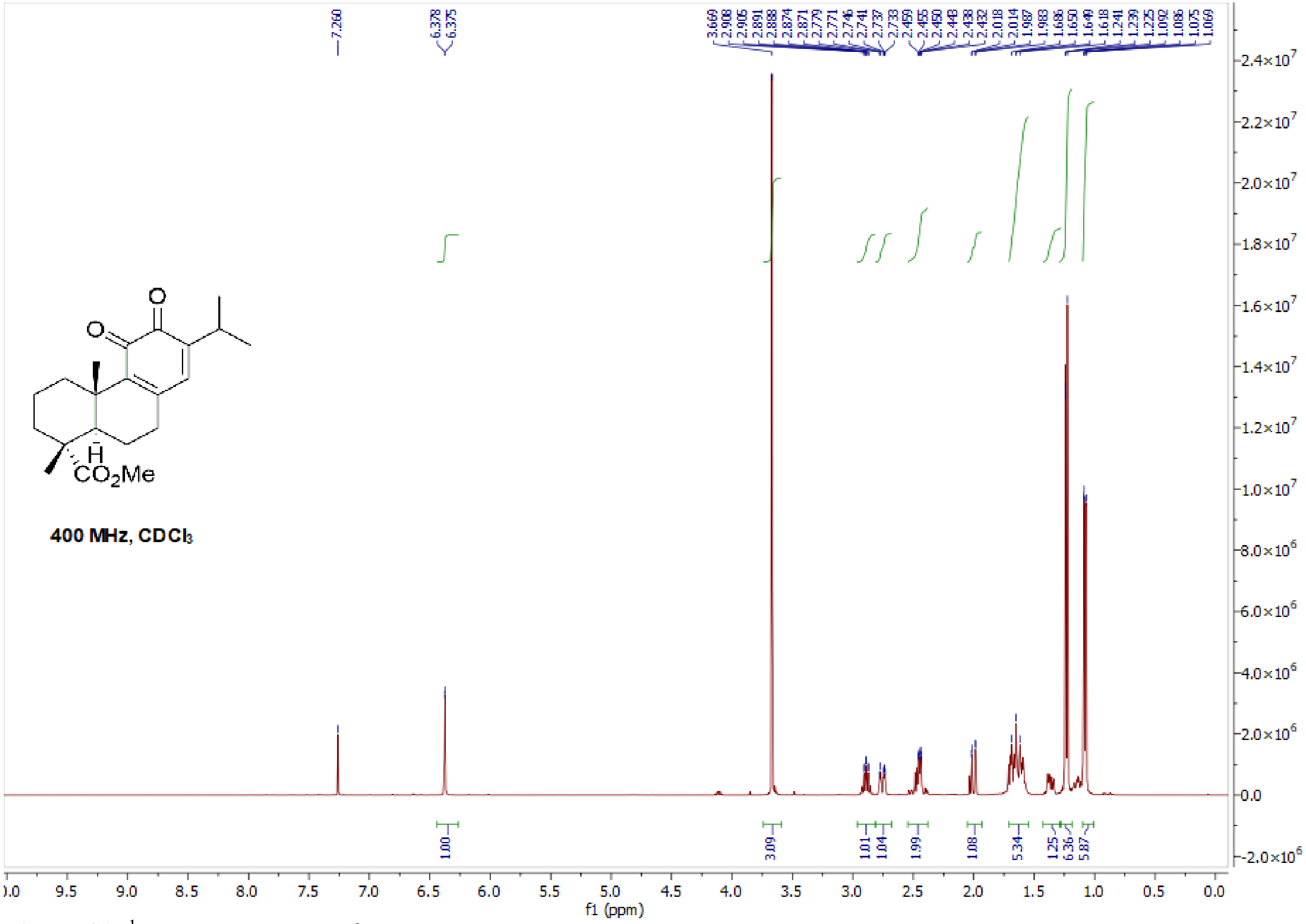

Figure S3. ^{1}H NMR spectrum of **7**.

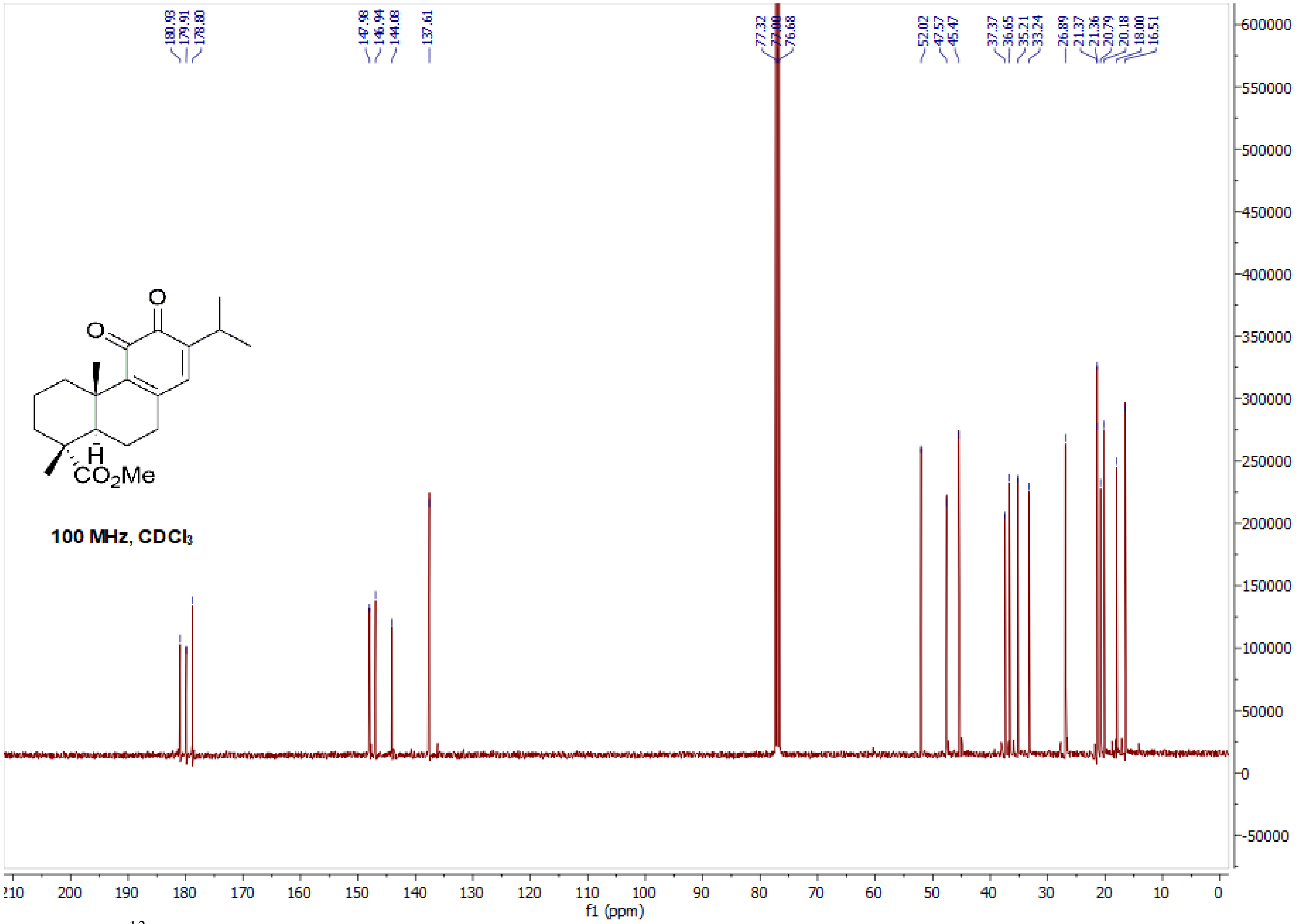

Figure S4. ^{13}C NMR spectrum of **7**.

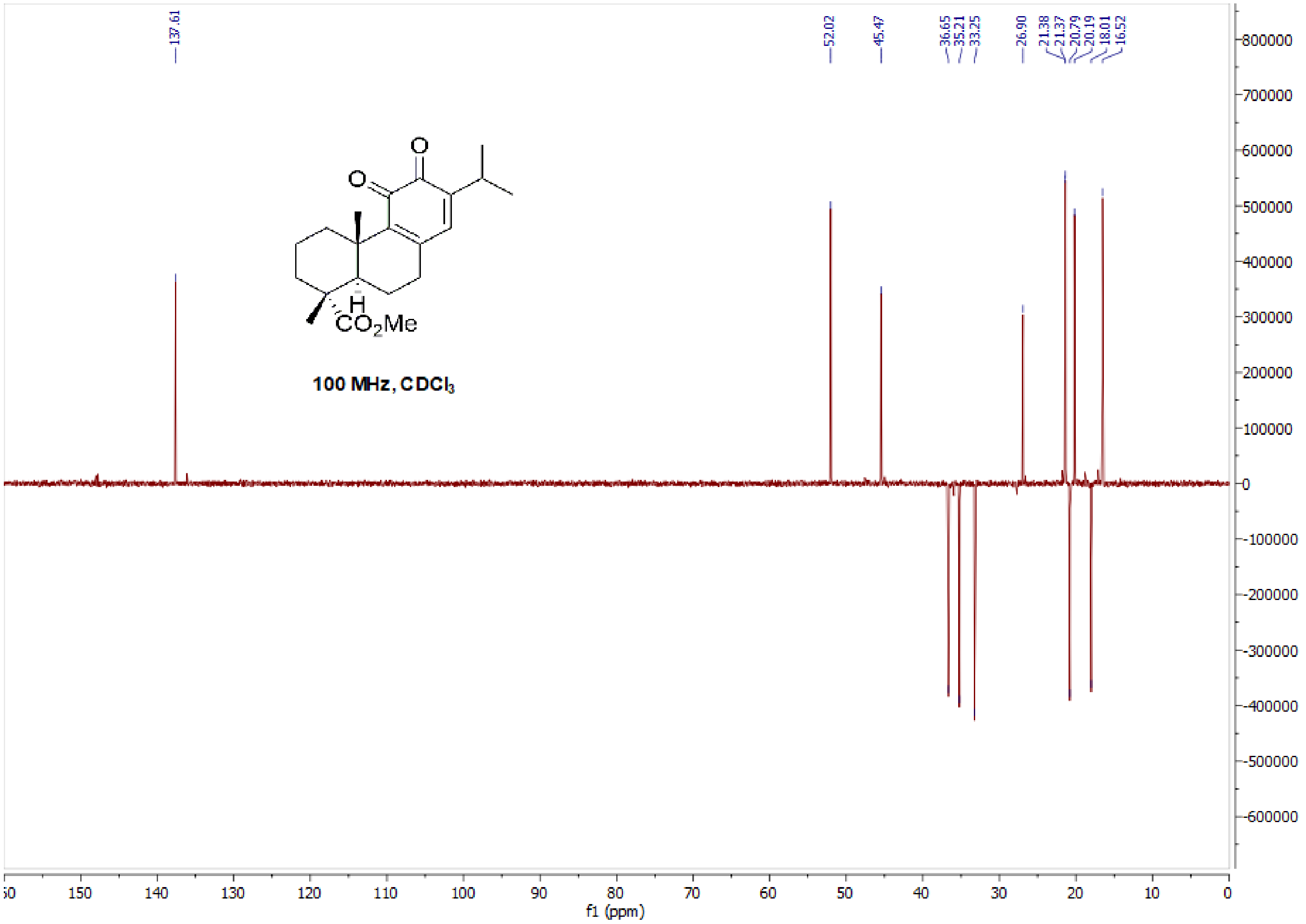

Figure S5. DEPT135 spectrum of **7**.

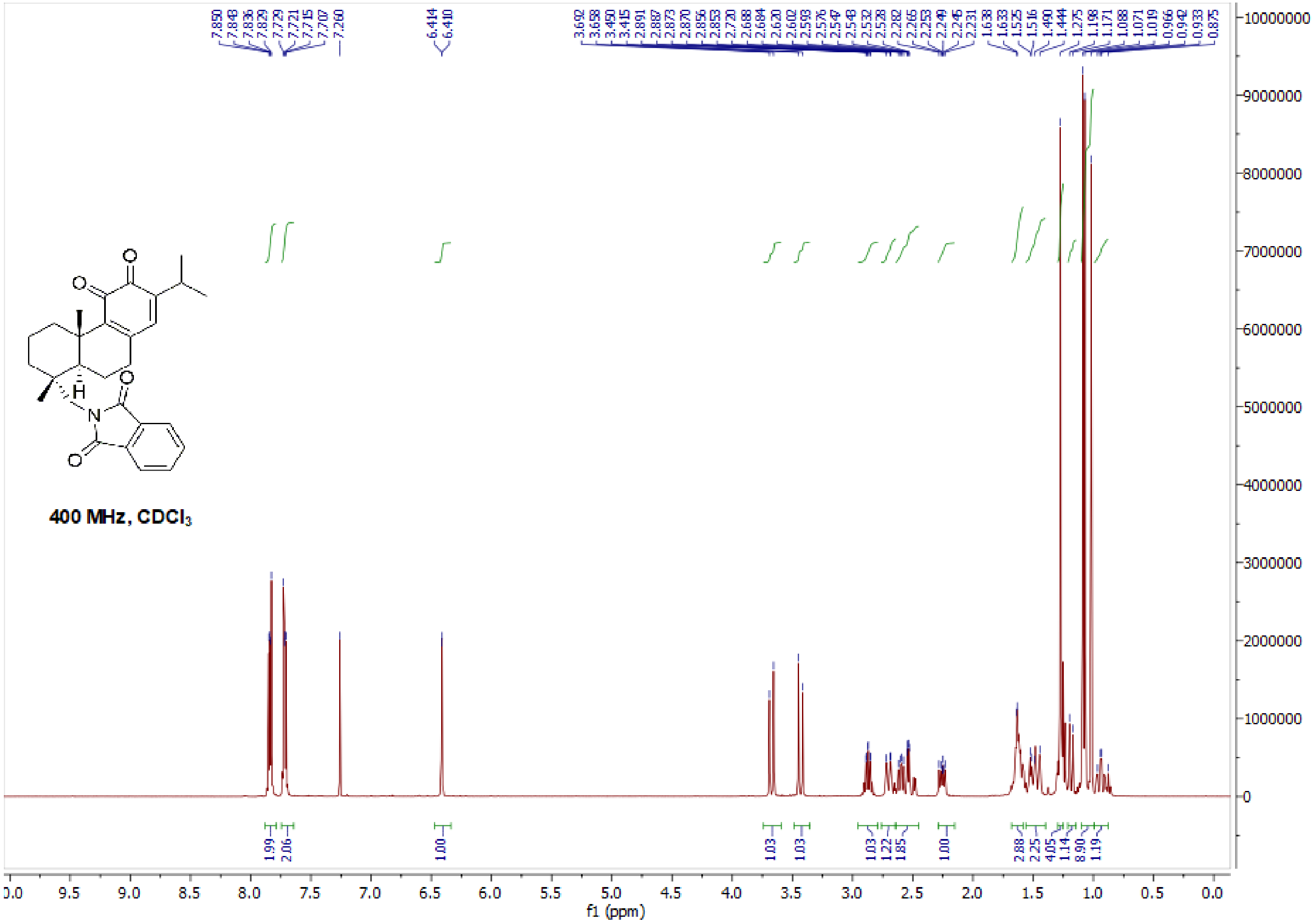

Figure S6. ^{1}H NMR spectrum of **8**.

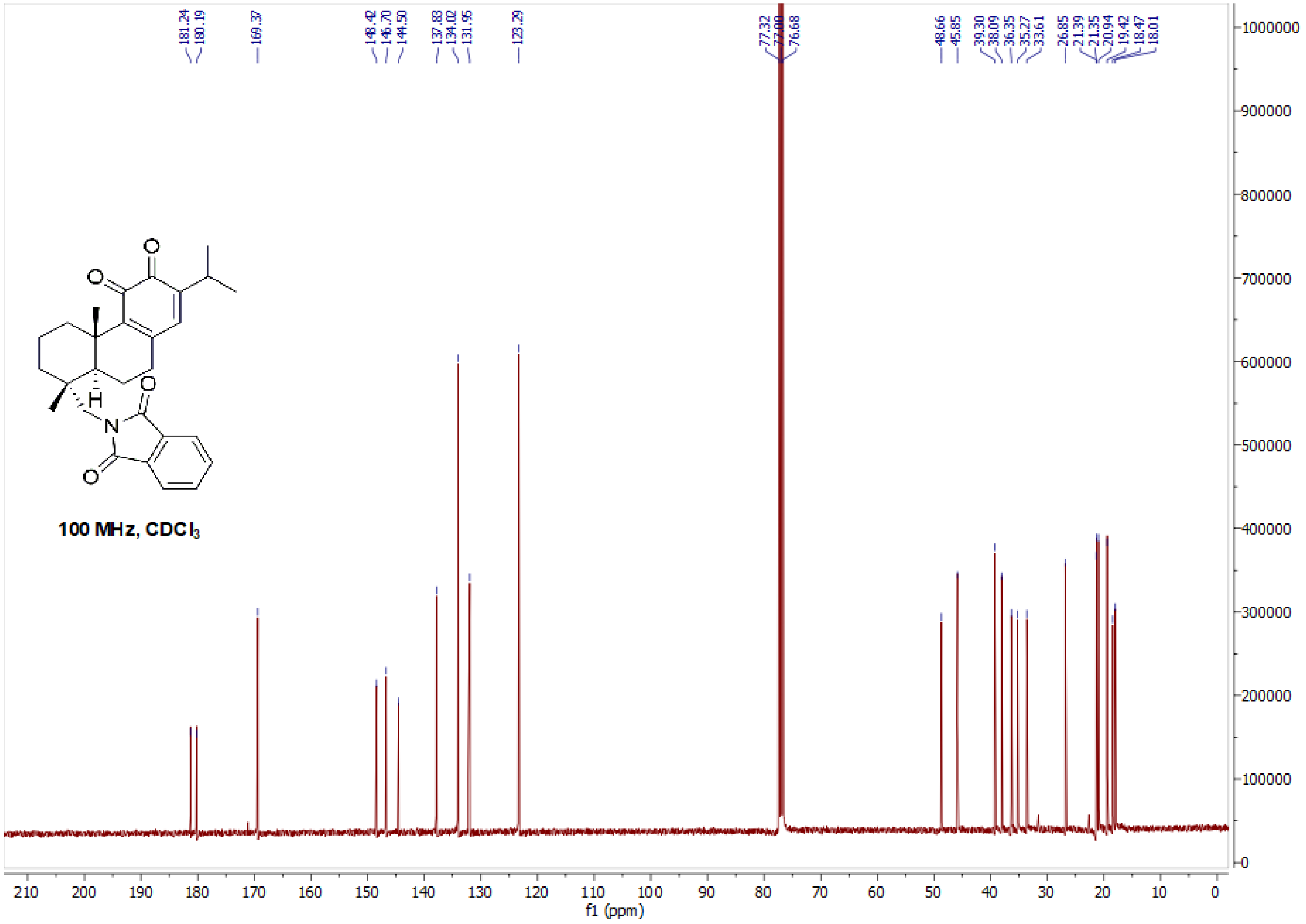

Figure S7. ^{13}C NMR spectrum of **8**.

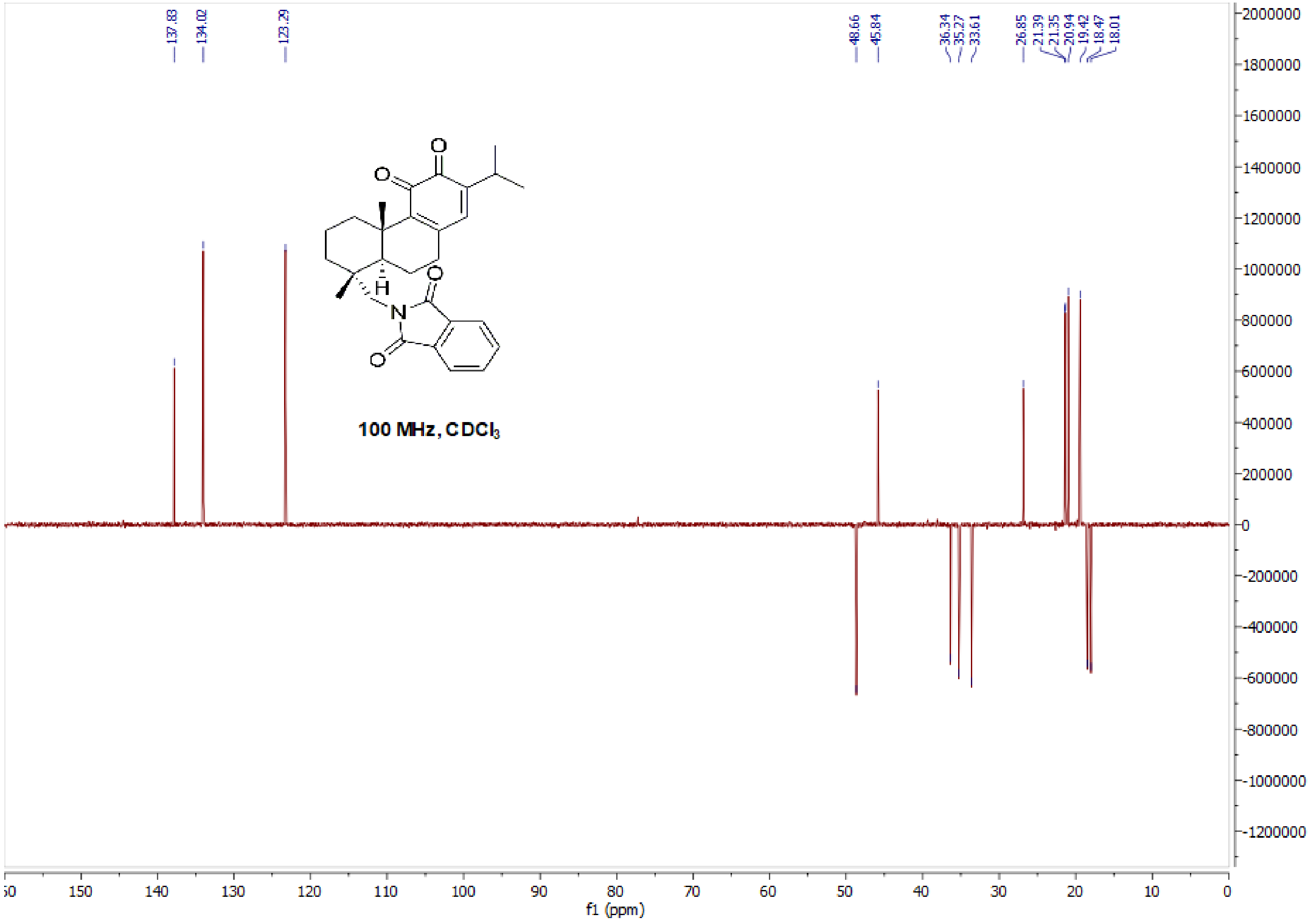

Figure S8. DEPT135 spectrum of **8**.

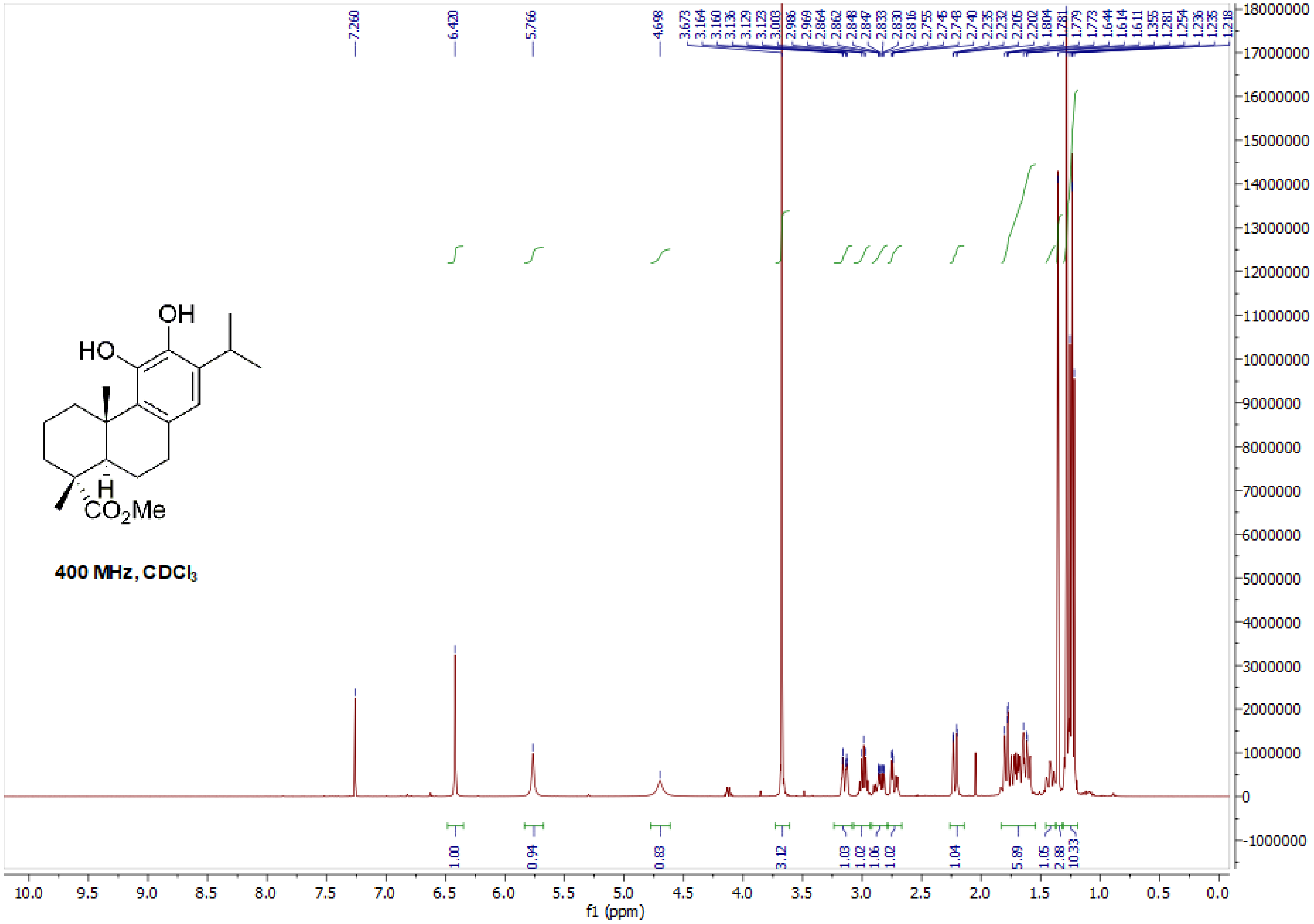

Figure S9. ^{1}H NMR spectrum of **9**.

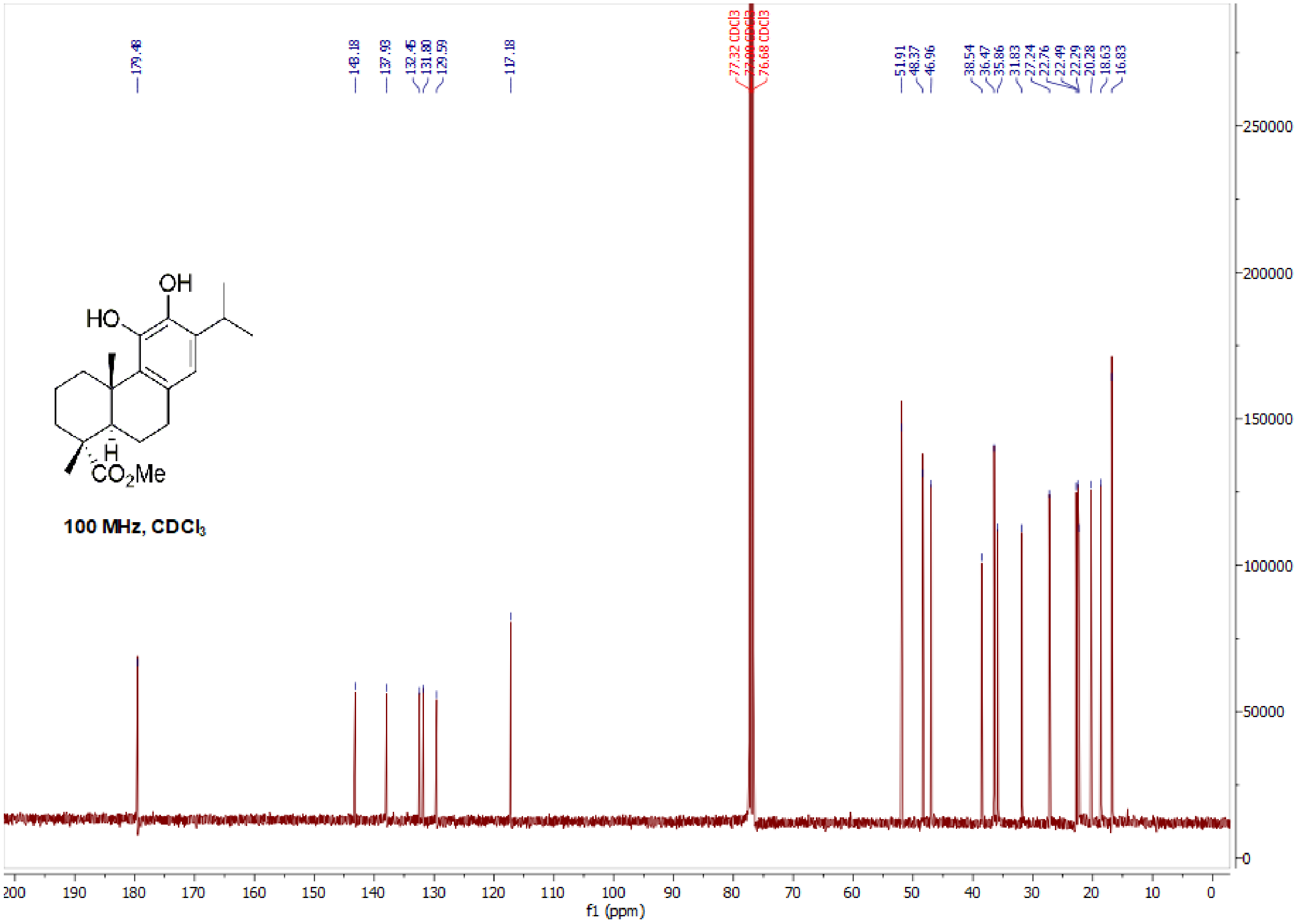

Figure S10. ^{13}C NMR spectrum of **9**.

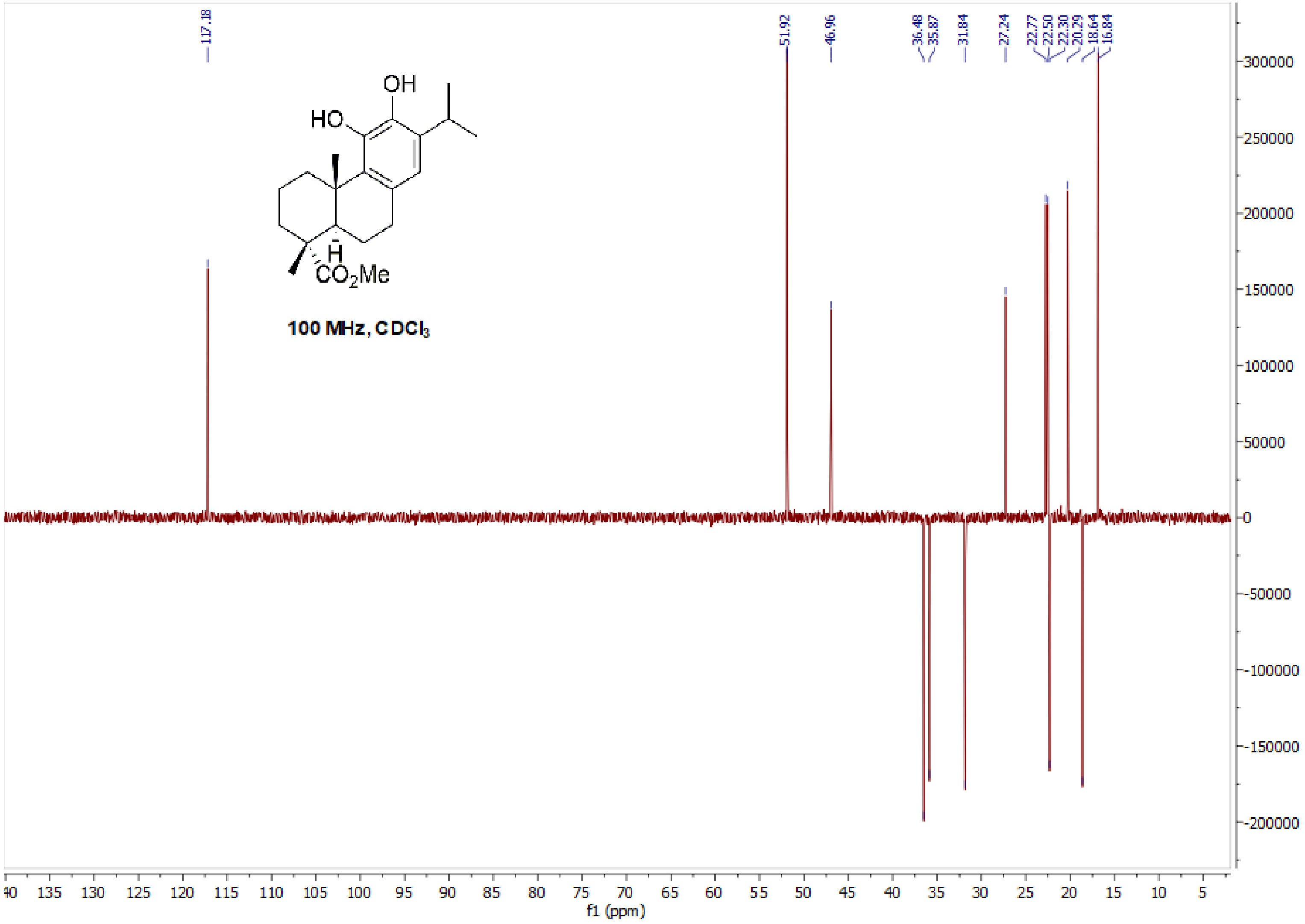

Figure S11. DEPT135 spectrum of **9**.

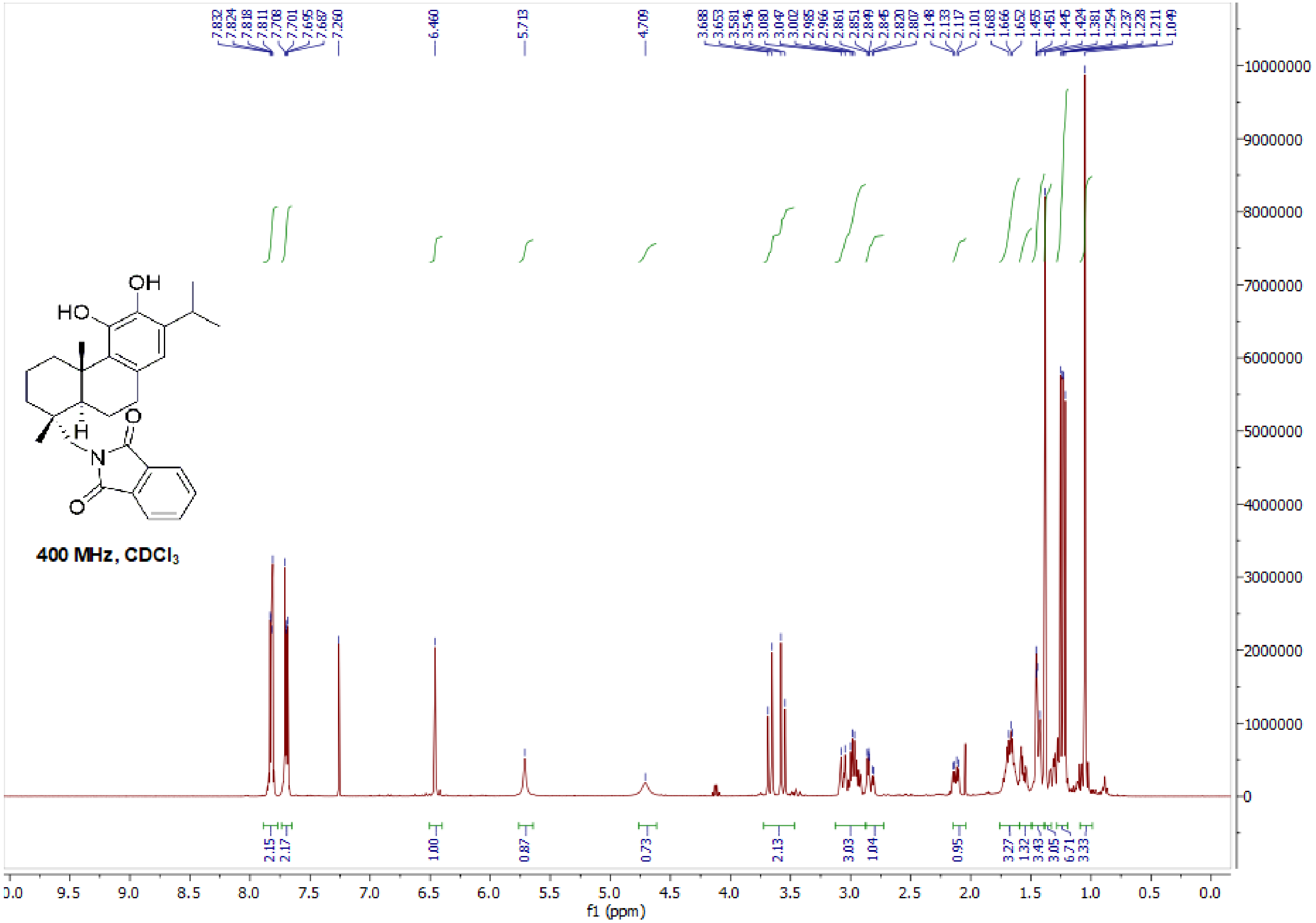

Figure S12. ^{1}H NMR spectrum of **10**.

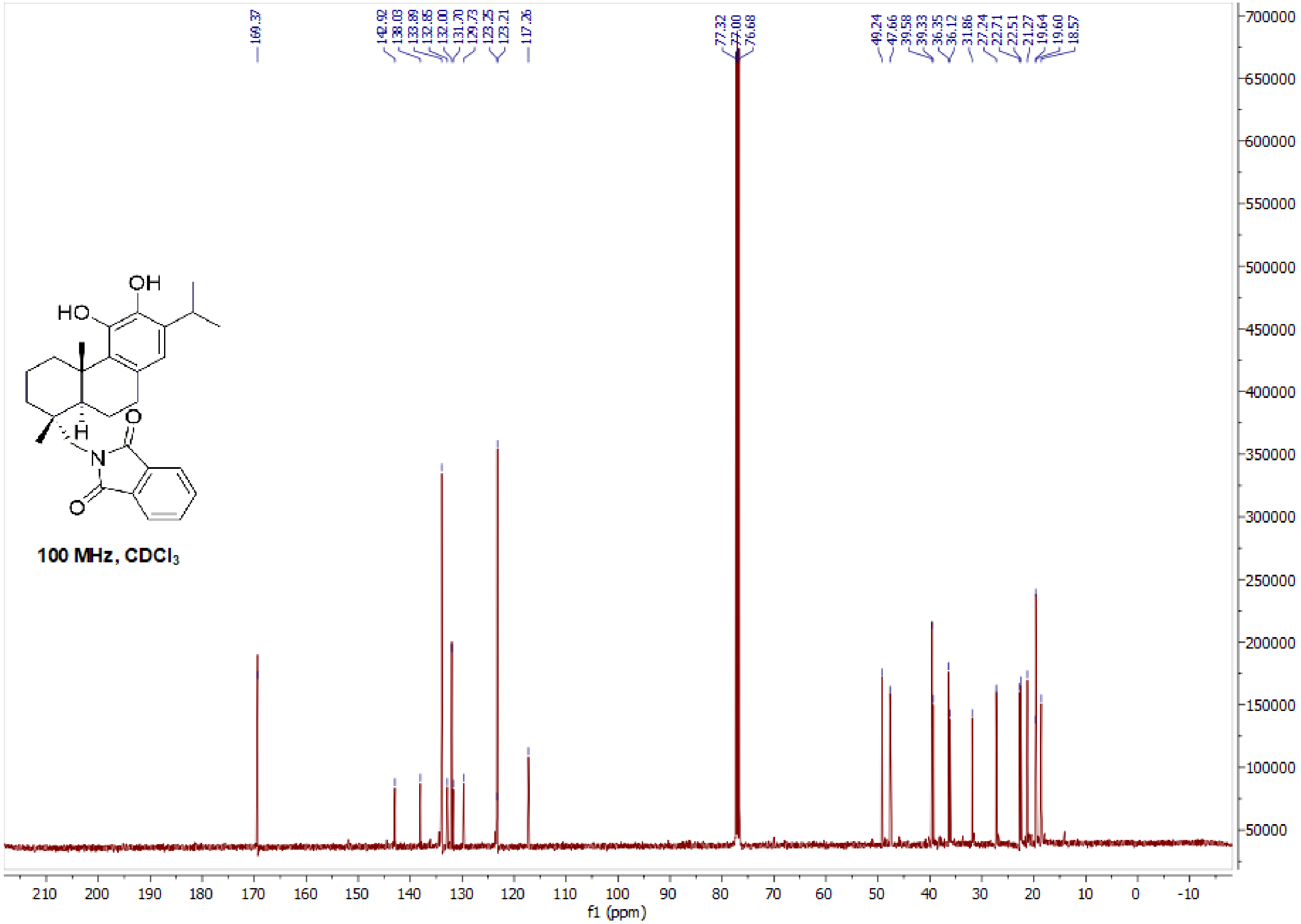

Figure S13. ^{13}C NMR spectrum of **10**.

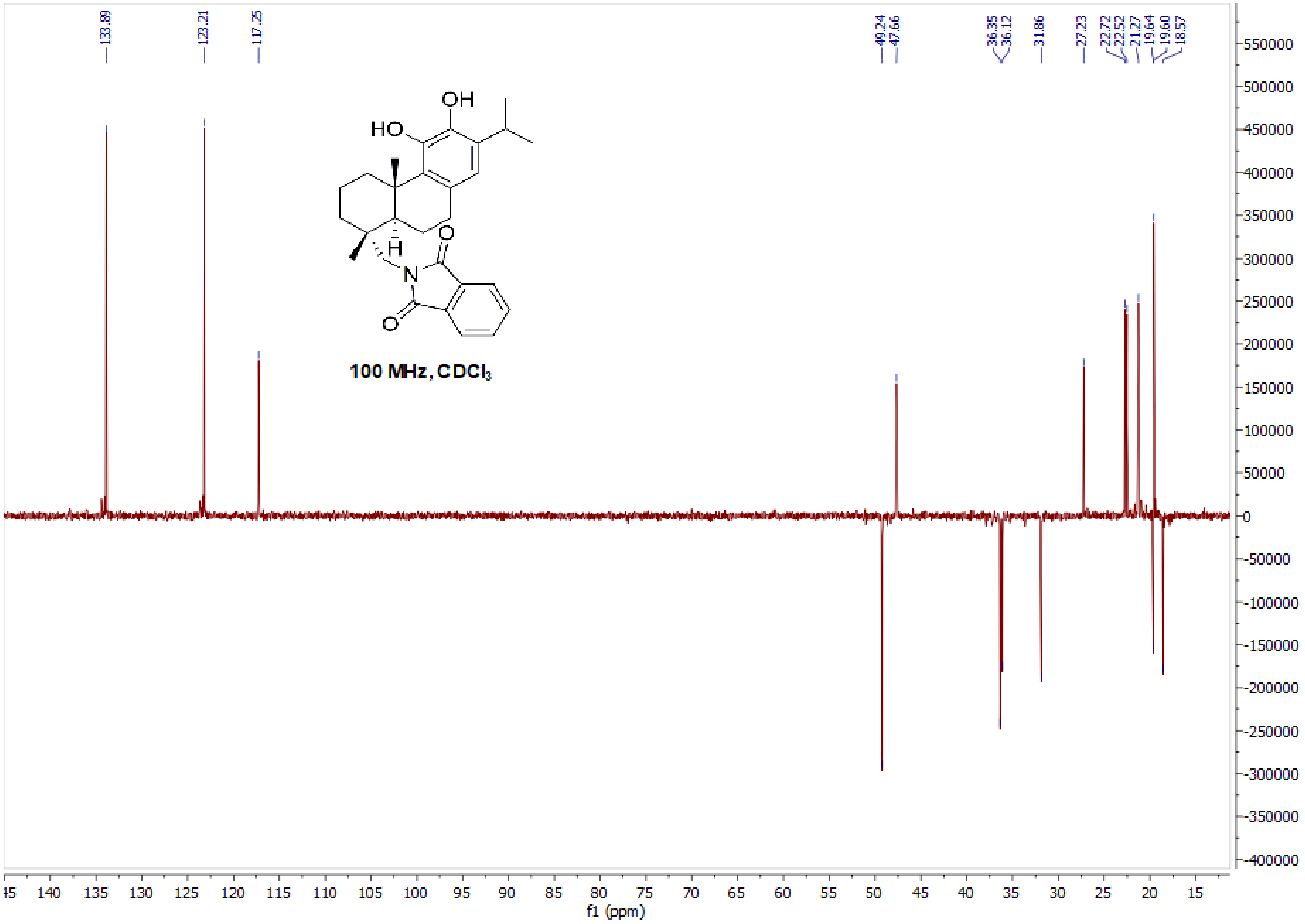

Figure S14. DEPT135 spectrum of **10**.
